# Softening due to Grain Boundary Cavity Formation and its Competition with Hardening in Helium Implanted Nanocrystalline Tungsten

**DOI:** 10.1038/s41598-018-20990-1

**Published:** 2018-02-13

**Authors:** W. Streit Cunningham, Jonathan M. Gentile, Osman El-Atwani, Chase N. Taylor, Mert Efe, Stuart A. Maloy, Jason R. Trelewicz

**Affiliations:** 1 Department of Materials Science and Chemical Engineering, Stony Brook University, Stony Brook, NY USA; 2 Materials Science and Technology Division, Los Alamos National Laboratory, Los Alamos, NM USA; 3 Fusion Safety Program, Idaho National Laboratory, Idaho Falls, ID USA; 4 Department of Metallurgical and Materials Engineering, Middle East Technical University, Ankara, Turkey

## Abstract

The unique ability of grain boundaries to act as effective sinks for radiation damage plays a significant role in nanocrystalline materials due to their large interfacial area per unit volume. Leveraging this mechanism in the design of tungsten as a plasma-facing material provides a potential pathway for enhancing its radiation tolerance under fusion-relevant conditions. In this study, we explore the impact of defect microstructures on the mechanical behavior of helium ion implanted nanocrystalline tungsten through nanoindentation. Softening was apparent across all implantation temperatures and attributed to bubble/cavity loaded grain boundaries suppressing the activation barrier for the onset of plasticity via grain boundary mediated dislocation nucleation. An increase in fluence placed cavity induced grain boundary softening in competition with hardening from intragranular defect loop damage, thus signaling a new transition in the mechanical behavior of helium implanted nanocrystalline tungsten.

## Introduction

Understanding the role of interfaces, such as grain boundaries, phase boundaries, etc., in the process of defect accumulation during irradiation represents a critical step forward in the design of radiation-tolerant nanomaterials^1–3^. Interfaces are well-known to act as sinks for the absorption of irradiation-induced defects where the nature of the interactions depend on the interfacial structure, proximity of the defects to the interface, and other intrinsic factors^4^. This mechanism for limiting non-recovered intergranular damage accumulation plays a significant role in nanostructured materials due to their large interfacial area per unit volume and has been explored in multilayer nanocomposites^5–10^ and nanocrystalline materials^11–16^. Enhanced radiation tolerance in the latter class of materials is often attributed to defect absorption by grain boundaries, which primarily accommodate interstitials as observed widely through displacement cascade simulations^17–20^. The atomic structure of the grain boundaries strongly influences their effective sink strength and involves both misorientation and boundary plane orientation^11,21–23^. This biased absorption of interstitials derives from their enhanced mobility and is accompanied by elevated vacancy concentrations at interstitial-loaded grain boundaries^20^. Subsequent emission of interstitial atoms from the boundaries leads to annihilation of vacancies through a recombination mechanism^24^, which can be exploited for limited irradiation damage in nanocrystalline materials.

Tungsten presents a unique opportunity to explore nanocrystalline grain structures for achieving enhanced radiation tolerance as it has emerged as a promising material for future fusion devices^25–27^. Plasma-facing materials will be subjected to extremely demanding operating environments involving high heat fluxes^28^, aggressive particle and neutron fluxes^29^, and high stresses^30^. Various forms of nanocrystalline tungsten have demonstrated potential improvements in the ductile-to-brittle transition temperature^31^, toughness^32–34^, and radiation tolerance^2,35–37^, where the latter has been postulated to derive from the aforementioned ability of grain boundaries to act as defect sinks^38^. Four stages have been identified in the defect accumulation trend: early and intermediate stages where the defect production rate increased with dose, a plateau stage attributed to defect saturation, and a final “recovery” stage defined by an apparent reduction in the defect density. Combined with high-resolution *in situ* imaging of defect interactions with grain boundaries, the recovery stage was attributed to synergistic defect coalescence and absorption by the boundaries^38^. These findings align with recent *in situ* analysis of denuded zone formation in nanocrystalline iron alloys under low-energy helium ion implantation^11^.

A number of damage mechanisms transpire in coarse-grained tungsten under helium ion implantation that depend on the incident ion energy and sample temperature, and have included defect loops^39,40^, helium bubbles^41–43^, and surface fuzz^44–47^. The presence of helium bubbles produces a surface hardening effect that is exacerbated by increasing fluence^48,49^. Helium bubble formation was also prevalent in ultrafine grained and nanocrystalline tungsten under a range of implantation conditions with large faceted bubbles preferentially forming in the grain boundaries at temperatures adequate for vacancy migration^50^. A grain size effect was noted with grains smaller than 40 nm virtually free of intragranular helium bubbles while grains larger than roughly 100 nm exhibited a uniform distribution of bubbles^35^. The drop in average bubble density was accompanied by a decrease in the average bubble size, which manifested as a reduced change in the grain volume particularly at higher fluence^51^. The suppression of intragranular volume changes combined with a seemingly greater fluence threshold for fuzz formation^52^ positions nanostructuring as a promising route for stabilizing tungsten against degradation under plasma conditions. However, a shift from dislocation to grain boundary mediated plasticity in nanocrystalline tungsten has been suggested to promote a softening effect coupled with intergranular fracture^53^, which could vitiate the potential benefits of nanostructuring for stabilization against bubble and fuzz formation.

In this study, the impact of helium ion implantation on the mechanical properties is explored in nanostructured tungsten containing a distribution of elongated nanocrystalline and ultrafine grains, referred to herein as nanocrystalline tungsten. Nanoindentation is employed to map the hardness and reduced modulus as a function of implantation temperature and fluence. We find that softening transpires across all implantation temperatures, which is correlated to the formation of bubbles/cavities in the grain boundaries with nuisances deriving from helium defects in the crystalline matrix. With increasing fluence, grain boundary cavities become faceted and increase in size, but are accompanied by the formation of defect loops within the interior of the grains. Hardening from intragranular defect loop damage thus competes with bubble/cavity induced grain boundary softening and the mechanisms underpinning this transition are discussed.

## Methods

Nanocrystalline tungsten containing a distribution of elongated nanocrystalline and ultrafine grains was produced by orthogonal machining as described in Efe *et al*.^37^. Samples were polished to a mirror-like finish using conventional metallurgical grinding and polishing techniques prior to ion implantation and further thinned for transmission electron microscopy (TEM) through electropolishing in a 0.05% NaOH solution. TEM was conducted using FEI-Tecnai-20 and FEI-Tecnai-G2-F30 microscopes with electron beam energies of 200 keV and 300 keV, respectively. Imaging was performed using Fresnel conditions to outline defects, which appear bright when under-focused and dark in an over-focused condition. Image processing was accomplished using the Image J software package^54^.

Helium ion implantation was conducted *ex situ* and *in situ* over a range of temperatures and fluences using two separate facilities. The Neutron Irradiated Material Ion Implantation Experiment (NIMIIX) at Idaho National Laboratory was employed for implanting samples *ex situ* at 30 °C (nominally room temperature) and 500 °C with 4 keV He^+^ ions introduced normal to the surface at a flux of 5.45 × 10^18^ He·m^−2^·s^−1^ to a fluence of 1.0 × 10^22^ He·m^−2^. For these conditions, the projected range of the helium ions was approximately 20 nm as calculated by the Stopping Range of Ions in Matter (SRIM) Monte Carlo computer code^55^, version 2013. Implantation to fluences over the range of 2.0 × 10^19^–3.2 × 10^20^ He·m^−2^ was accomplished *in situ* in the JEOL JEM-2000FX TEM at the Microscope and Ion Accelerator for Materials Investigations (MIAMI) facility at the University of Huddersfield^56^. Specifically, samples were implanted at 30 and 500 °C with 4 keV He^+^ to fluences of 2.0 × 10^19^ and 2.0 × 10^20^ He·m^−2^, and at 950 °C with 2 keV He^+^ to 3.6 × 10^19^ and 3.2 × 10^20^ He·m^−2^. *In situ* implantations were performed under a constant flux of 3.2 × 10^16^ He·m^−2^·s^−1^ with an incident angle of 30° relative to the surface normal, which augmented the projected damage range via SRIM to approximately 10 nm.

Nanoindentation was conducted at room temperature using a Hysitron TS75 Triboscope lateral force transducer coupled to a Bruker Dimension Icon atomic force microscope with a z-axis load and depth resolution of 0.1 μN and 0.2 nm, respectively. The indentation samples were roughly 3 mm diameter discs that were first electropolished and followed by helium ion implantation, which was conducted uniformly over their entire sample surface; the only additional surface preparation employed prior to nanoindentation was cleaning with organic solvents. Thinning via electropolishing was highly concentrated to the center of the sample and produced a small hole with electron-transparent regions around its edges (i.e., within a few tens of microns) that were leveraged for TEM imaging. The rest of the disc was only cleaned through electropolishing and its overall thickness remained close to the initial thickness of approximately 100 μm. Indentation measurements were performed on regions sufficiently removed from the central hole and thus representative of a “bulk” measurement relative to the maximum indentation depth of approximately 250 nm.

A diamond Berkovich probe with a radius of 50 nm was employed with the area function calibrated on fused silica over a contact depth range of 25–200 nm. To minimize thermal drift, the probe tip was placed in contact with the specimen surfaces for at least one hour prior to testing. Each sample was indented at a constant indentation strain rate of 0.5 s^−1^ and indentation loads were selected to limit the total contact depth to <85 nm; further details on the significance of constant indentation strain rate can be found elsewhere^57^. Instrumental drift was characterized during the linear unloading segment using a 10 s hold at 10% of the maximum load, and a minimum of 10 indents with negligible drift were used in quantifying the hardness and reduced modulus via the Oliver and Pharr method^58^.

## Results

### Nanoindentation of Helium Ion Implanted Nanocrystalline Tungsten

**Figure 1:**
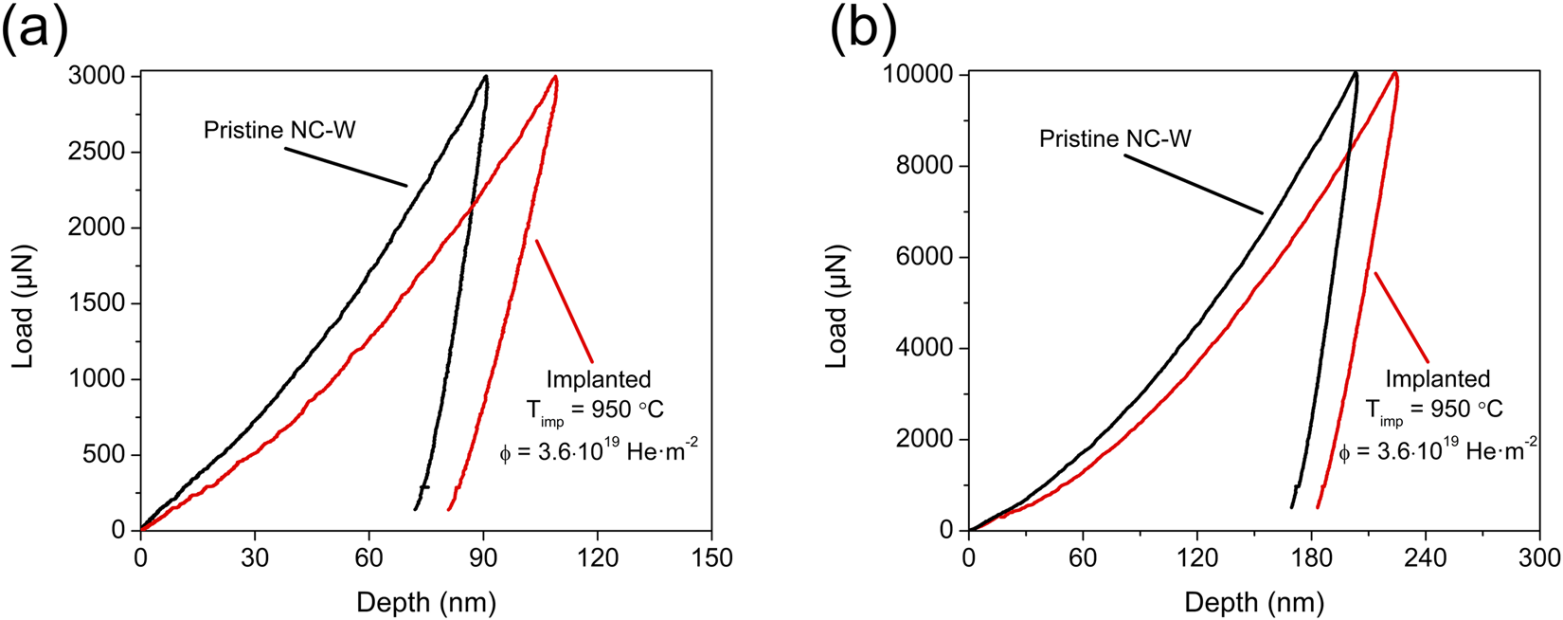
Representative load-displacement curves for nanocrystalline tungsten in pristine and helium ion implanted states indented to maximum loads of (**a**) 3 mN and (**b**) 10 mN. The implanted sample exhibited a shift in the load-displacement curves to larger indentation depths indicative of a reduction in hardness, which was more prominent at the shallower depths under the 3 mN load.

Representative load-displacement curves are shown in Fig. 1 for pristine nanocrystalline tungsten and following implantation at 950 °C to a fluence of 3.6 × 10^19^ He·m^−2^. The implanted sample exhibited a shift in the load-displacement curve to larger indentation depths relative to pristine nanocrystalline tungsten that will ultimately manifest as a reduction in the hardness. This shift was more pronounced for the lower indentation load of 3 mN in Fig. 1a and diminished with an increase in load to 10 mN in Fig. 1b. The depth dependence of the shift in the loading curves indicates that softening was most prevalent near the surface in the range of the projected damage region. Specifically, the data acquired at 3 mN derived from sampling depths of ≤85 nm and an increase in load to 10 mN more than doubled the residual depth. Although the plastic zone size at both maximum indentation loads will exceed the predicted damage range of 10–20 nm from SRIM, the stress field is far more localized beneath the indenter tip at the lower indentation depths produced by the 3 mN load. Given that yielding in crystalline metals follows a maximum shear stress criterion, plasticity will be initiated more locally beneath the indenter tip in a region much smaller than the final plastic zone size as the tip is driven into the material. Subsequent property measurements thus employed a maximum load of 3 mN, which adequately captures the impact of the ion damaged zone while simultaneously minimizing other potential effects that can enhance measurement error at extremely shallow indentation depths such as surface roughness or breakdown of the tip area function.

**Figure 2:**
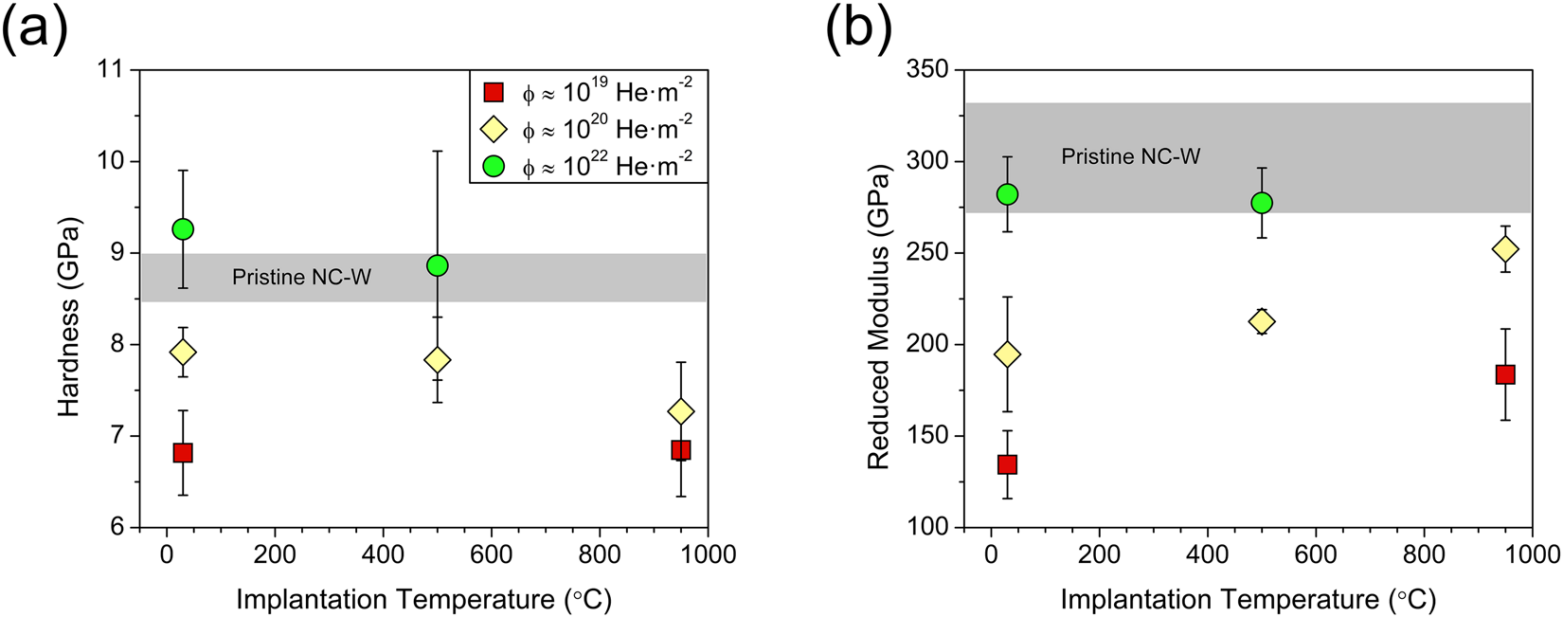
(**a**) Indentation hardness and (**b**) reduced modulus as a function of implantation temperature for a maximum indentation load of 3 mN. Data points are indexed based on fluence with the legend shown in (**a**), and ranges are included for pristine nanocrystalline tungsten.

Hardness and reduced modulus are shown as a function of implantation temperature in Fig. 2a and b, respectively, with data points indexed by fluence (Φ). In the pristine condition, the nanocrystalline tungsten samples exhibited a hardness of 8.7 ± 0.3 GPa and reduced modulus of 302 ± 30.0 GPa, and the corresponding ranges are shown in the figures for reference. Room temperature implantation to a fluence of 3.6 × 10^19^ He·m^−2^ produced a significant reduction in the hardness and modulus relative to the pristine nanocrystalline tungsten sample. An order of magnitude increase in fluence to 3.2 × 10^20^ He·m^−2^ was accompanied by an increase in both properties as compared with the lower fluence condition, which was further accentuated at a fluence of 1.0 × 10^22^ He·m^−2^. In fact, the hardness exceeded the pristine nanocrystalline tungsten sample at this fluence and thus resembled behavior characteristic of classical irradiation hardening. Consequently, softening that dominated at low fluences transitioned to hardening with increasing fluence, indicating that competing mechanisms were activated by different microstructural damage states.

Analogous scaling behavior was evident at 500 °C where initial softening succumbed to hardening with increasing fluence. The degree of hardening and softening was comparable to the room temperature property shifts, which suggests that similar mechanisms were activated across this temperature range. At 950 °C, property scaling with fluence was consistent with the trends observed for the lower temperature implantations, i.e. both the hardness and reduced modulus initially exhibited a sharp decline relative to the pristine nanocrystalline tungsten sample, which was followed by an increase with increasing fluence. Two differences are noted in the property shifts at 950 °C relative to the behavior of the samples implanted at room temperature. First, the recovery in the hardness with increasing in fluence was not as extensive in Fig. 2a. Second, the drop in modulus from the pristine nanocrystalline tungsten sample in Fig. 2b was less severe especially at 3.6 × 10^20^ He·m^−2^. This apparent dependence on implantation temperature suggests that the mechanisms accommodating plasticity are fundamentally altered by the defect microstructures formed at 950 °C as compared with lower temperature implantations.

**Figure 3:**
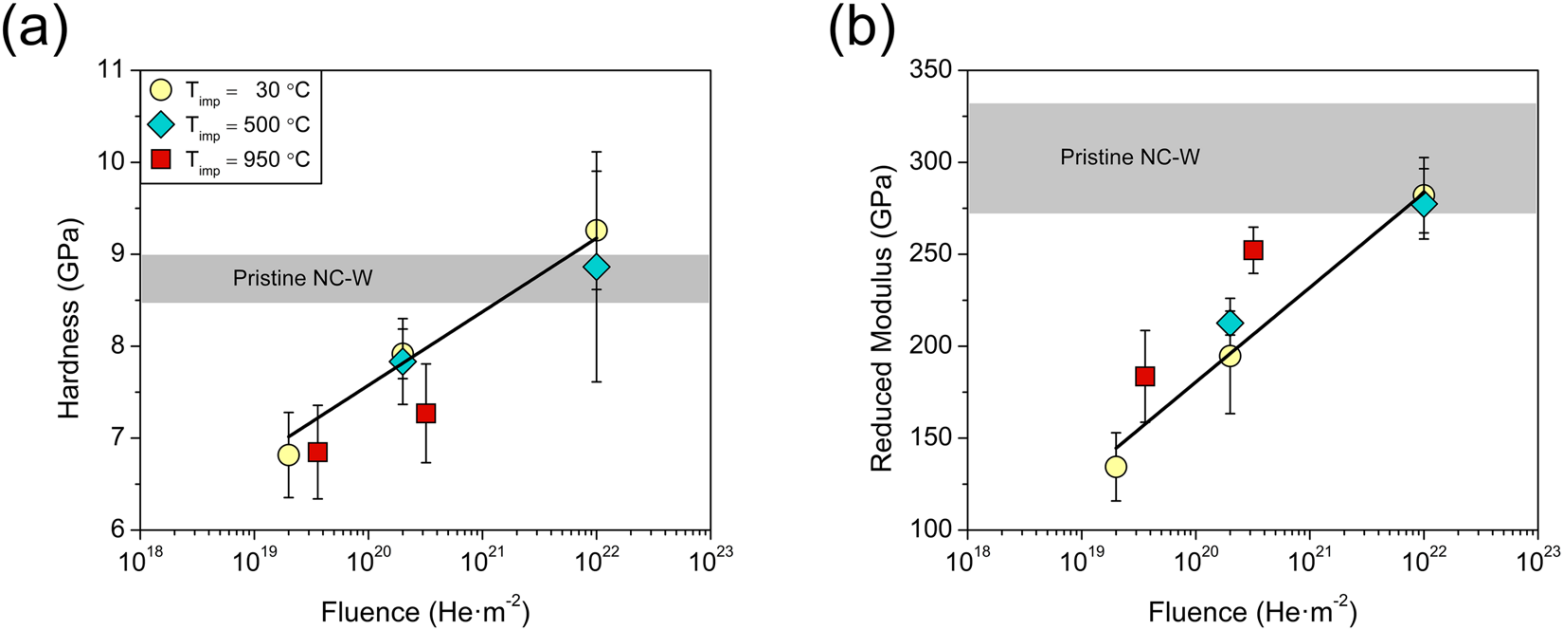
(**a**) Indentation hardness and (**b**) reduced modulus as a function of fluence. Data is indexed based on implantation temperature and collapses to a single linear trend for T_imp_ ≤ 500 °C. Properties of the samples implanted at 950 °C deviated from this trend line, indicating that different mechanisms governed the transition from softening to hardening with increasing implantation temperature.

To substantiate the seemingly disparate behavior at different implantation temperatures, we replotted the hardness and reduced modulus as a function of fluence in Fig. 3a and b, respectively. Data points are indexed based on implantation temperature (T_imp_) and exhibited a continuous increase with fluence across all temperatures as expected from the results in Fig. 2. However, Fig. 3 demonstrates that both the hardness and modulus scaled linearly with fluence, and the data for T_imp_ ≤ 500 °C collapsed onto a signal linear trend line. The consistent scaling behavior for T_imp_ ≤ 500 °C supports a common mechanism governed the transition from softening to hardening with increasing fluence over this temperature range. Conversely, the properties of the samples implanted at 950 °C convincingly deviated from the linear scaling behavior at lower implantation temperatures as captured by the hardness vales falling below the linear trend line in Fig. 3a, which was accompanied by a consistently greater modulus in Fig. 3b. The emergence of different scaling behavior at 950 °C indicated that hardening with fluence has an inherent dependence on the implantation temperature, and its microstructural underpinnings are explored in the next section.

### Characterization of Damage Microstructures

**Figure 4:**
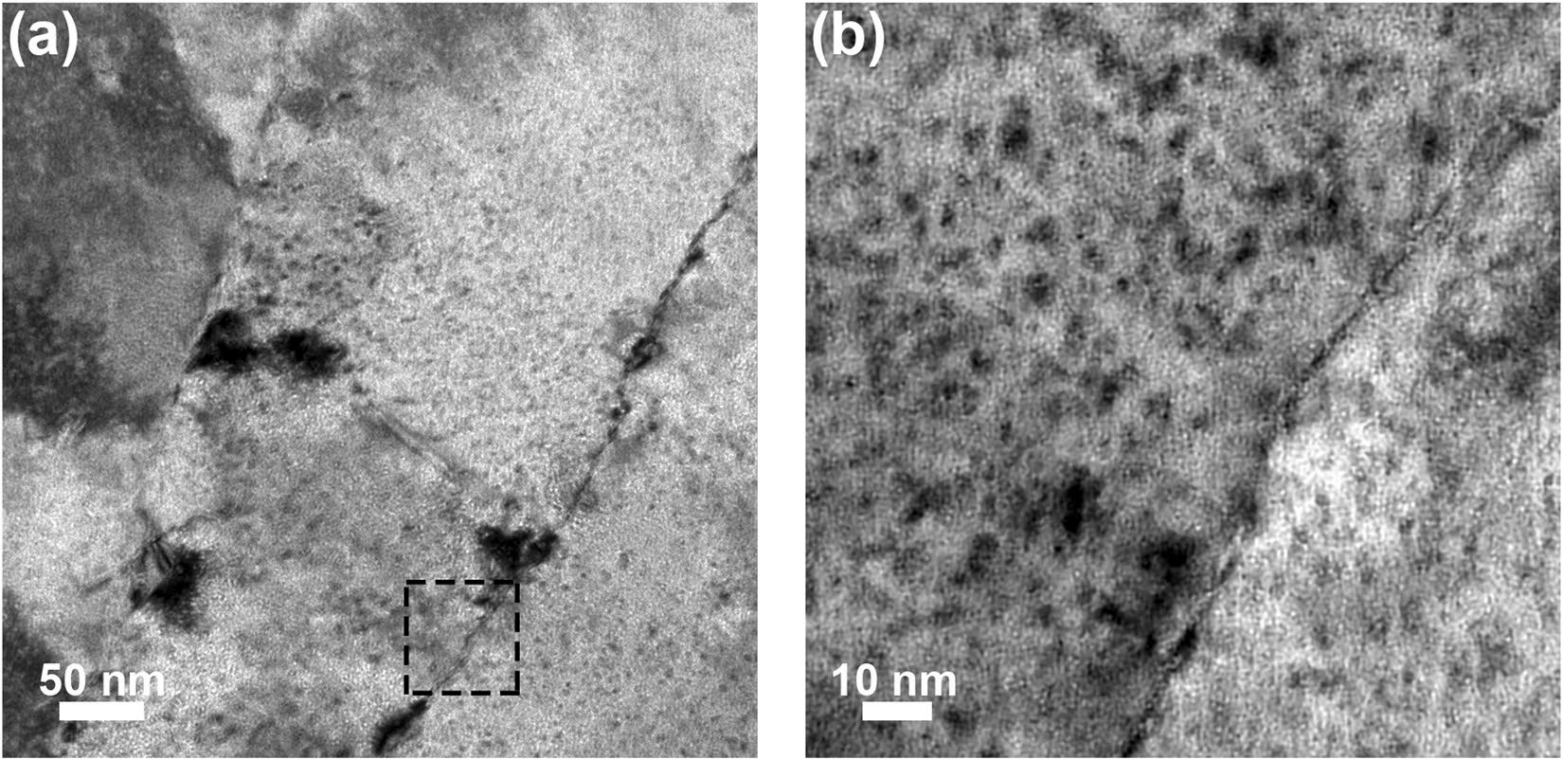
Bright-field TEM image of the nanocrystalline tungsten sample implanted at room temperature to a fluence of 2.0 × 10^20^ He·m^−2^. The region denoted in (**a**) is shown magnified and under-focused in (**b**) and demonstrates a uniform distribution of bubbles in the damage microstructure.

**Figure 5:**
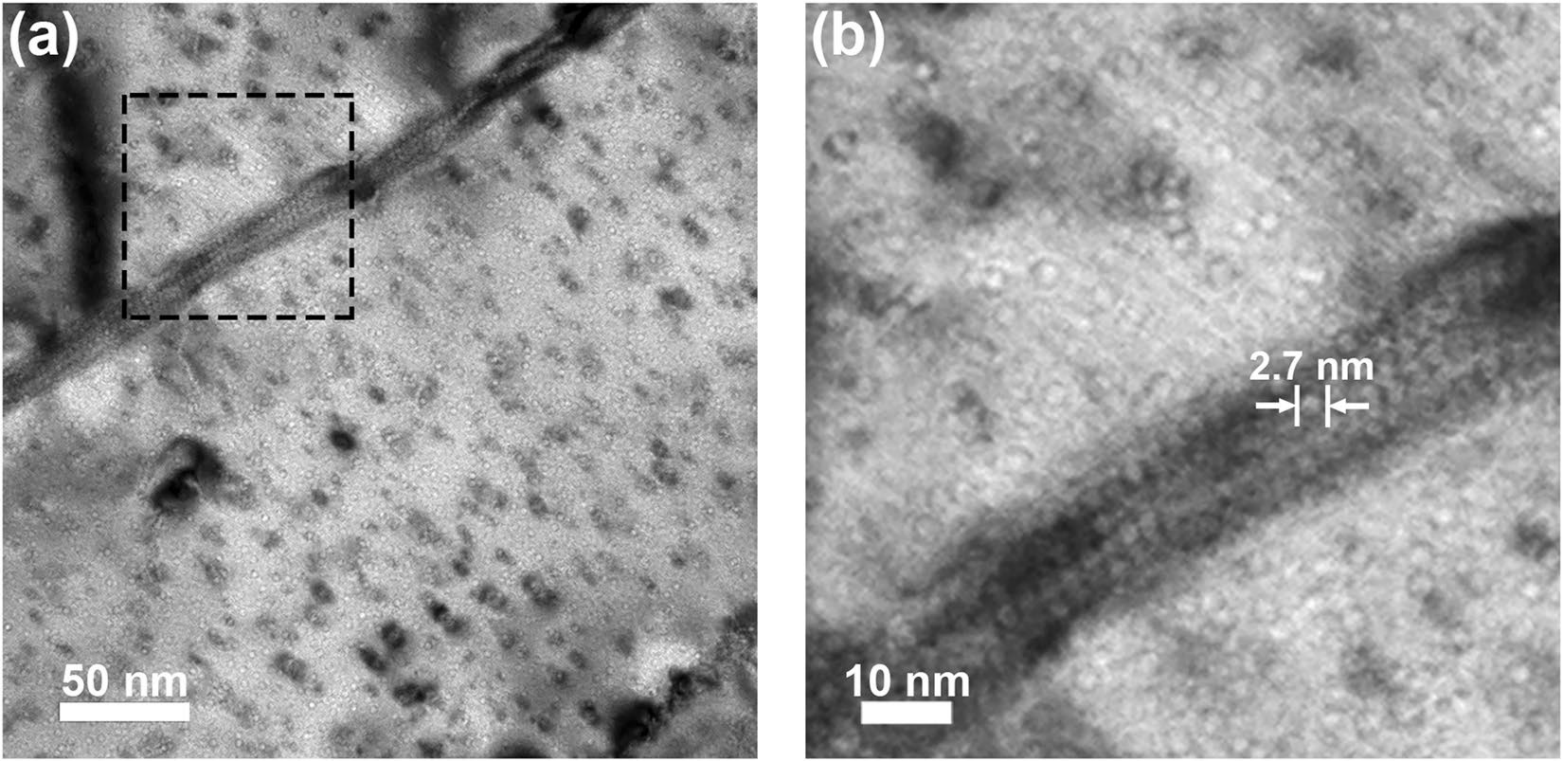
Bright-field TEM image of the nanocrystalline tungsten sample implanted at 500 °C to a fluence of 2.0 × 10^20^ He·m^−2^. The region denoted in (**a**) is shown magnified in (**b**) and demonstrates a uniform distribution of bubbles between the grain interior and grain boundary.

Prior work has demonstrated the formation of helium bubbles and cavities in grain boundaries of ultrafine grained and nanocrystalline tungsten particularly at temperatures adequate for vacancy migration^50,51^. In this section, damage microstructures produced by the implantation conditions used in this study are explored with particular focus on the size and distribution of helium bubbles as a function of implantation temperature and fluence. A description of the pristine tungsten microstructures can be found in literature for both the as-prepared condition^37,50^ and following heat treatment at 950 °C^35^. The bright-field image in Fig. 4a was acquired on the nanocrystalline tungsten sample implanted at room temperature to a fluence of 2.0 × 10^20^ He·m^−2^. A magnified and under-focused image of the inset containing the grain boundary is shown in Fig. 4b, which demonstrates the presence of helium bubbles distributed uniformly throughout the microstructure. The average bubble size was generally <1 nm, and it was thus difficult to ascertain distributions that could be delineated for the grain interior and grain boundaries. An increase in temperature to 500 °C for a nominally equivalent fluence produced the damage microstructure shown in Fig. 5a with a magnified image of the inset containing the grain boundary shown in Fig. 5b. A uniform distribution of helium bubbles was evident in both the grain interiors and grain boundaries and consistent with the damage microstructure formed during room temperature implantation. However, the bubbles were discernibly larger with average size of 2.7 ± 0.3 nm as illustrated in Fig. 5b, which enabled distributions to be quantified for the grain interior and grain boundary regions and revisited in the next section.

**Figure 6:**
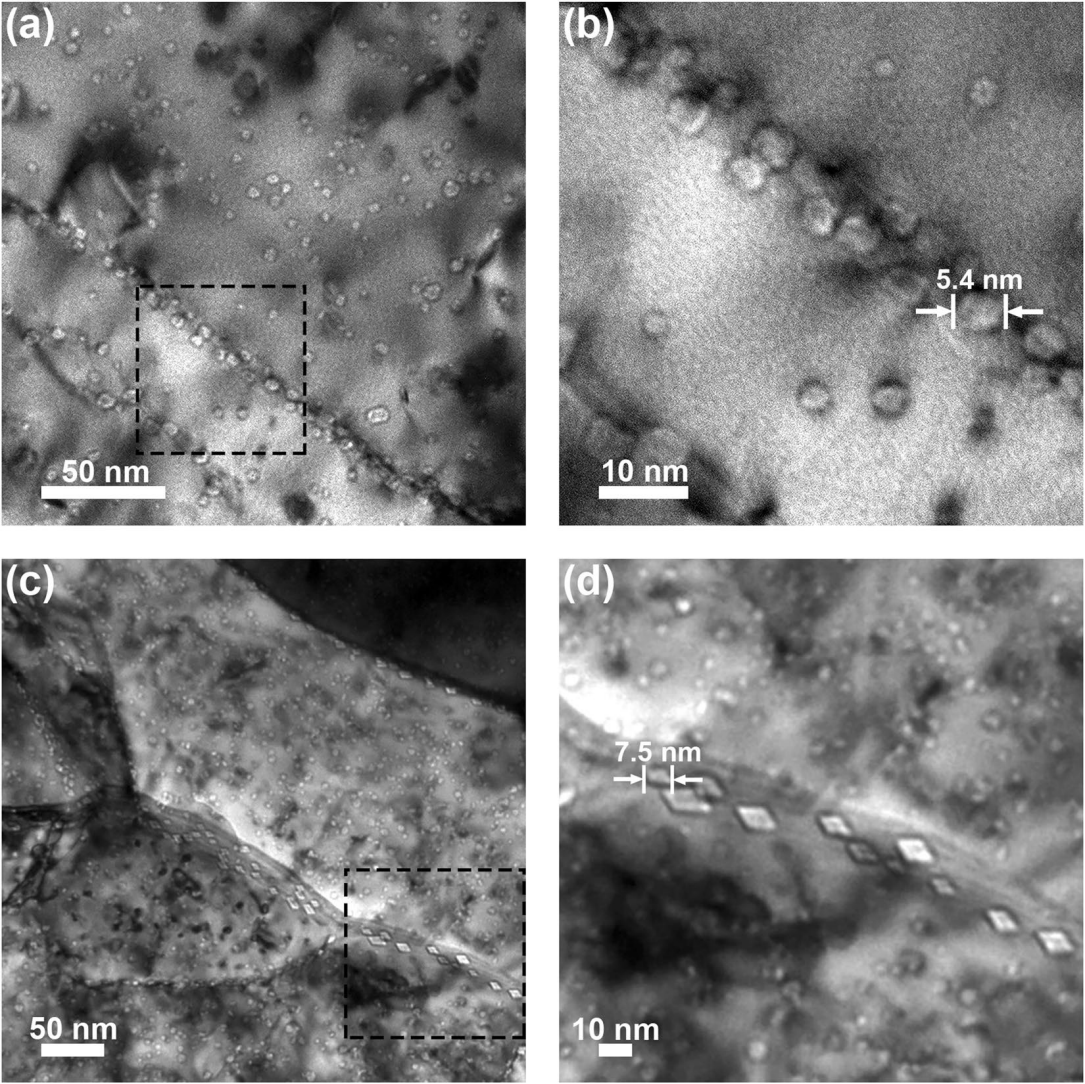
Bright-field TEM images of the damage microstructures resulting from implantation at 950 °C to a fluence of (**a**) 3.6 × 10^19^ He·m^−2^ with a magnified image of the inset shown in (**b**) and (**c**) 3.2 × 10^20^ He·m^−2^ with a magnified image of the inset shown in (**d**). Preferential aggregation of spherical cavities in the grain boundaries at the lower fluence transitioned to larger faceted cavities with increasing fluence.

**Figure 7:**
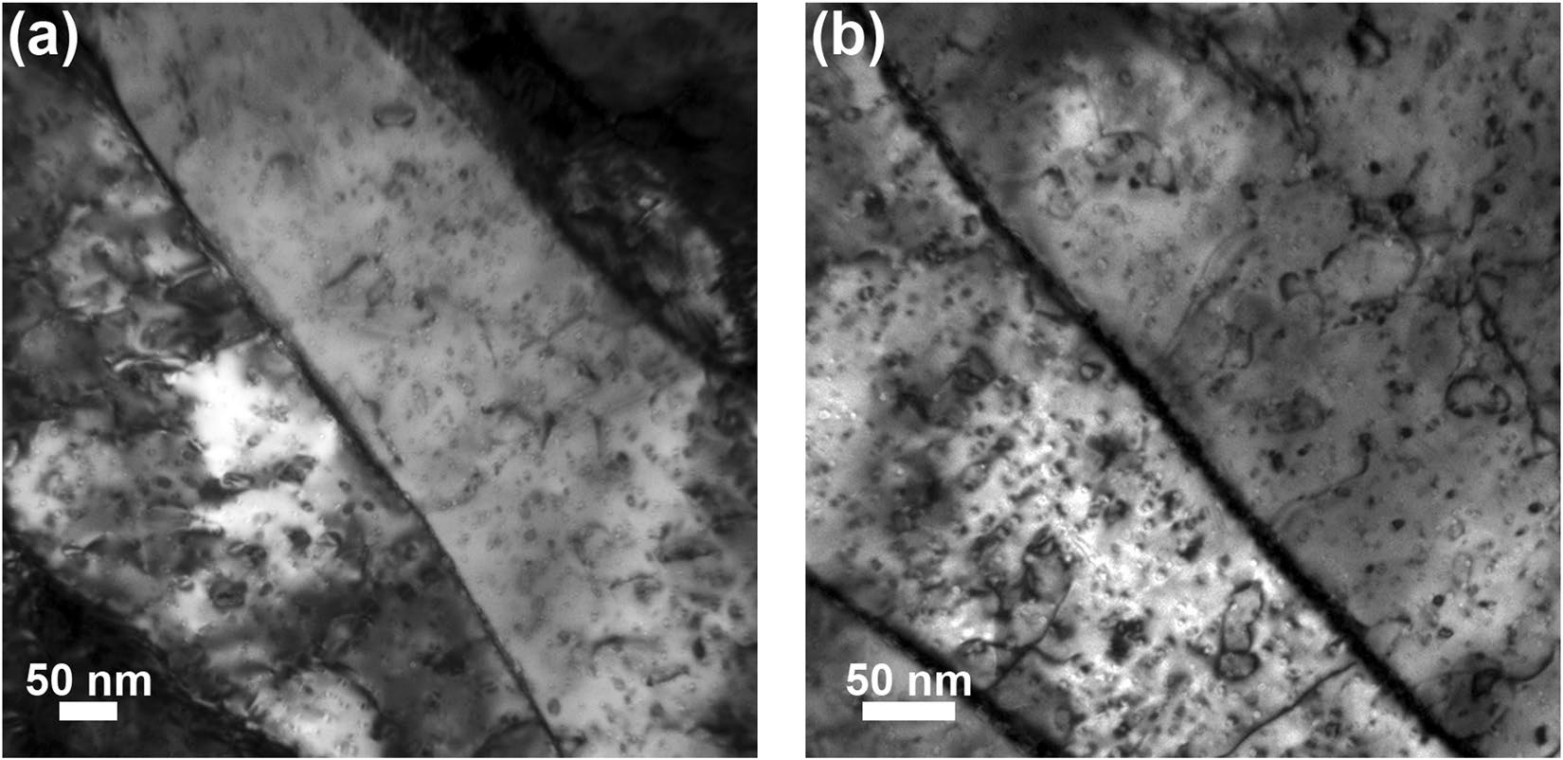
Bright-field TEM images of intragranular defect loop damage in the nanocrystalline tungsten samples implanted at 950 °C to fluences of (**a**) 3.6 × 10^19^ and (**b**) 3.2 × 10^20^ He·m^−2^. The increase in fluence was accompanied by an increase in the number density of defect loops that formed within the crystalline matrix.

Implantation of the sample at 950 °C to a fluence of 3.6 × 10^19^ He·m^−2^ produced the damage microstructure in Fig. 6a with a magnified image of the inset containing the grain boundary shown in Fig. 6b. Although helium defects were apparent in the interior of the grains, preferential aggregation of these defects transpired at the grain boundary. The average defect size increased to 5.4 ± 1.1 nm as illustrated in Fig. 6b, and indicative of a transition to the formation of cavities as noted in literature^59^. The damage microstructure following implantation at 950 °C to the higher fluence of 3.2 × 10^20^ He·m^−2^ is shown in Fig. 6c. A complex damage state was observed that included helium bubbles/cavities distributed throughout the microstructure with differing morphologies. While defects in the grain interiors retained their spherical character, cavities occupying the boundaries appeared faceted and were markedly larger with an average size of 7.5 ± 1.3 nm as illustrated in Fig. 6d. Intragranular defect loop damage absent at the lower fluence as shown in Fig. 7a also emerged with the increase in fluence to 3.2 × 10^20^ He·m^−2^ in Fig. 7b. With increasing fluence, additional helium atoms become available for the formation of helium-vacancy complexes, which also serve as nucleation sites for the formation of dislocation loops^60^. The presence of larger helium cavities coupled with the onset of intragranular defect loop damage was thus consistent with defect formation mechanisms in tungsten during helium ion implantation^50^.

## Discussion

The damage microstructures in helium implanted nanocrystalline tungsten are qualitatively summarized as follows: samples implanted at room temperature and 500 °C to a fluence of 2.0 × 10^20^ He·m^−2^ exhibited a uniform bubble distribution within the microstructure, and increasing the implantation temperature to 950 °C promoted preferential defect alignment in the grain boundaries with sizes indicating a transition to cavity formation. An increase in fluence particularly at 950 °C produced large faceted cavities in the grain boundaries, which were accompanied by smaller cavities and defect loop damage within the grains. These findings are consistent with literature results that have demonstrated uniform helium bubble formation in the crystalline matrix at temperatures where vacancy mobility is limited and a transition to preferential grain boundary occupation at temperatures adequate for vacancy migration^42,50,61,62^.

**Figure 8:**
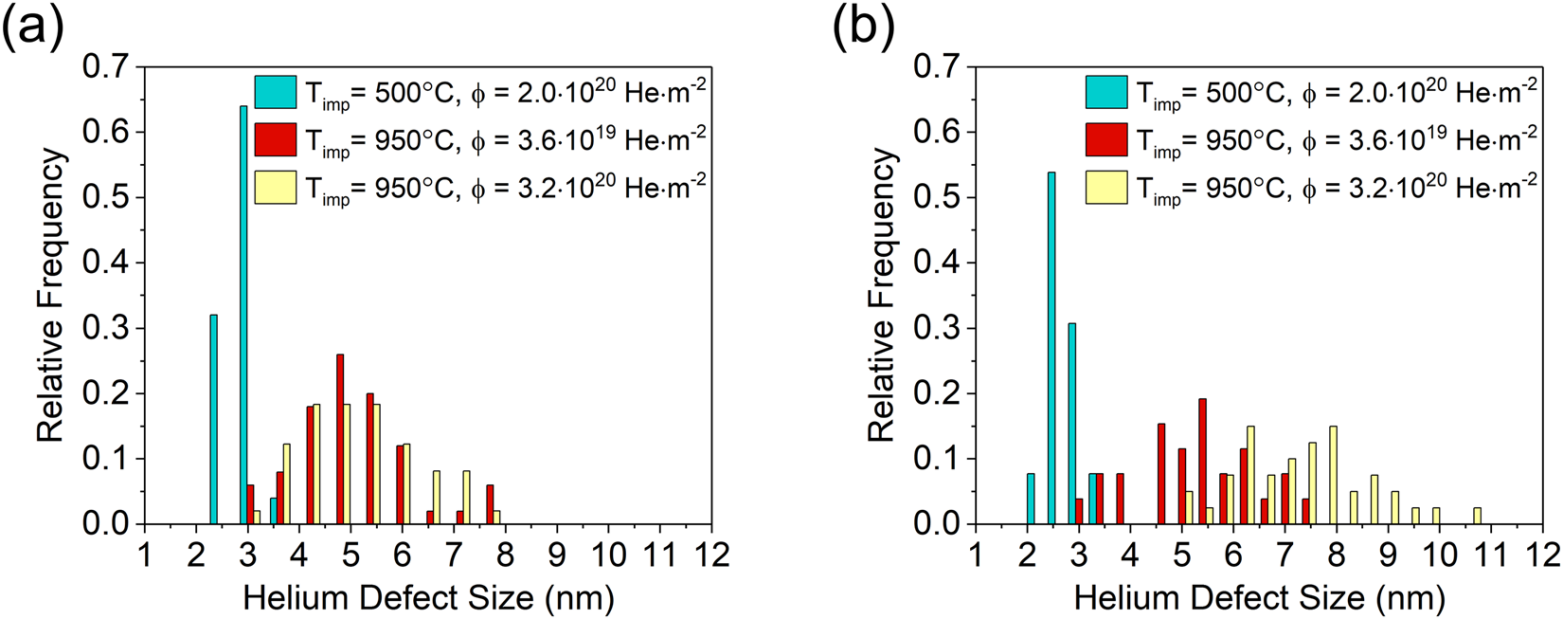
Defect size distributions for (**a**) grain interiors and (**b**) grain boundaries as a function of the implantation conditions including temperature and fluence. Bubble and cavity sizes scaled primarily with temperature in the grain interior whereas fluence also impacted the cavity size distribution in the grain boundaries.

Correlations between the various defect microstructures and measured mechanical properties were explored by quantifying helium defect size distributions in the grain interior and grain boundary regions in Fig. 8a and b, respectively. At implantation temperatures up to 500 °C, helium defects were distributed uniformly between the grain interior and grain boundaries as illustrated in Fig. 8 for the sample implanted at 500 °C. The primary difference between the defects formed over this temperature range was their average size: room temperature implantation produced bubbles with diameters of <1 nm while implantation at 500 °C promoted an increase in bubble size up to 3.5 nm. The reduction in hardness and modulus upon implantation at temperatures up to 500 °C can thus be attributed to the formation of helium bubbles in the microstructure, and the extent of this degradation was independent of the bubble size over the range of <1–3.5 nm. Implantation at 950 °C produced a conspicuous increase in the size of the helium defects occupying the interior of the grains as captured by the shift of the distribution to larger defect sizes in Fig. 8a, which also transpired in the grain boundaries at the lower fluence of 3.6 × 10^19^ He·m^−2^. Although the average cavity size was comparable in both regions under these conditions, the grain boundaries contained a larger number density relative to the grain interiors as shown in Fig. 6a. The degree of softening in these samples was consistent with the lower temperature implantations in spite of larger helium defects being aggregated to grain boundaries.

The implications of bubble and cavity formation for the mechanical behavior of nanocrystalline tungsten can be understood using insights from molecular dynamics (MD) simulations of deformation in BCC nanocrystalline metals. Grain size strongly dictates the deformation behavior of nanocrystalline metals and at the finest grain sizes approaching the amorphous limit, deformation has been shown to involve mechanisms such as grain boundary sliding in iron^63^ and grain rotation facilitated by local atomic reconfigurations within the grain boundaries in tantalum^64^. Grain boundary sliding was also identified as a dominant mechanism accommodating plasticity in nanocrystalline tungsten with a grain size of approximately 5 nm^53^. The addition of hydrogen and helium atoms with concentrations spanning the range of 0.5–15 at. % promoted strain localization in the grain boundaries, which was accompanied by a discernible drop in flow stress. An increase to intermediate nanocrystalline grain sizes in the range of 10–100 nm has been demonstrated to produce a shift to grain boundary mediated dislocation plasticity in nanocrystalline BCC metals^64,65^. This transition to discrete dislocation plasticity with an increase in grain size from MD simulations was consistent with experiments that revealed enhanced quasi-static mechanical properties in tungsten containing elongated nanocrystalline grains.

The nanocrystalline tungsten samples explored in the present study contained a distribution of grain sizes generally greater than 30 nm. Based on the deformation behavior of nanocrystalline BCC metals highlighted above, plasticity is likely accommodated by grain boundary mediated dislocation plasticity in our samples. Softening due to helium ion implantation was correlated to the formation of helium bubbles and cavities in both the crystalline matrix and grain boundaries. Voids are known to act as stress concentrations that reduce the energetic barrier for dislocation nucleation from grain boundaries^66^ as well as accelerate grain boundary mediated deformation processes^67^. Consequently, the observed softening effect in nanocrystalline tungsten following helium implantation can be explained by the presence of bubbles and cavities reducing the activation barrier for dislocation nucleation at grain boundaries and in turn, the onset of plasticity. We finally note that the increase in modulus that accompanied softening particularly in the sample implanted at 950 °C to a fluence of 3.6 × 10^19^ He·m^−2^ was instead attributed to the reduced number density of cavities forming within the crystalline matrix (i.e. grain interiors) due to their aggregation in the grain boundaries.

An increase in fluence to 10^20^ He·m^−2^ across all implantation temperatures produced an important competing effect where the hardness and modulus increased relative to the nanocrystalline tungsten samples irradiated at 10^19^ He·m^−2^, but still remained decidedly below the range for the pristine sample. At the higher fluence, the cavity size distribution in the grain boundaries shifted to distinguishably larger values in Fig. 8b, which was absent in the grain interiors and underscores the role of grain boundaries in defect accumulation under helium ion implantation. However, intragranular cavity formation was also accompanied by the emergence of defect loop damage within the interior of the grains in Fig. 7b. While the presence of larger cavities in the grain boundaries suggests that the mechanical properties should be further diminished with increasing fluence, the complex damage microstructure leads to a confluence of mechanisms that places softening from bubbles and cavities in competition with hardening from defect loop damage, which was further exacerbated at the highest fluence of 1.0 × 10^22^ He·m^−2^.

Hardening with increasing fluence can be attributed to the combination of dislocation loops and intragranular helium defects inhibiting dislocation slip^39,40^. This particular effect was illustrated by Orowan^68^ where the change in shear stress, Δτ, is given by:

1 $${\rm{\Delta }}\tau =\alpha Gb\sqrt{Nd}$$

with α representing the defect barrier strength, G the shear modulus, b the Burgers vector, and N and d the number density and diameter of the defects (cavities or loops), respectively. In the high fluence samples, increases in the loop density was qualitatively evident in Fig. 7b. Consequently, hardening in the grain matrices was exacerbated following Eq. (1) and offset the contribution from softening due to cavity loaded grain boundaries augmenting the onset of plasticity. In light of these competing mechanisms, the dominant effect will depend on the defects formed under various implantation conditions and their interaction with grain boundaries, which will be amplified in nanocrystalline tungsten due to the inherently high interfacial volume fraction. Our findings thus provide a foundation for further exploration of these competing mechanisms in nanocrystalline metals with particular focus on implantation conditions that produce cavity loaded grain boundaries collectively with intragranular defect damage.

## Conclusions

The mechanical behavior of helium implanted nanocrystalline tungsten was explored via nanoindentation with hardness and reduced modulus mapped as a function of implantation temperature and fluence. Softening was apparent across all implantation temperatures and attributed to bubble/cavity loaded grain boundaries suppressing the activation barrier for grain boundary mediated dislocation plasticity. Conversely, the scaling of the modulus with implantation temperature was ascribed to the reduced number density of cavities remaining within the crystalline matrix due to their aggregation into larger cavities with preferential alignment at the grain boundaries. While both hardness and modulus scaled with fluence for all implantation temperatures, the results for implantation up to 500 °C collapsed onto a single linear trend. The properties of the samples implanted at 950 °C, which is adequate for migration of helium-vacancy complexes, deviated from this linear scaling behavior due to the formation of larger cavities and their preferential aggregation to the grain boundaries. Despite the different scaling relationships, hardening was correlated to the formation of intragranular defect loop damage with increasing fluence. The complex damage state of helium ion implanted nanocrystalline tungsten thus produced a confluence of mechanisms that places softening due to grain boundary cavity formation in competition with hardening from intragranular defect loop damage.
